# Elevational gradient of Hemiptera (Heteroptera, Auchenorrhyncha) on a tropical mountain in Papua New Guinea

**DOI:** 10.7717/peerj.978

**Published:** 2015-06-02

**Authors:** Maxime Le Cesne, Stephen W. Wilson, Adeline Soulier-Perkins

**Affiliations:** 1 Muséum National d’Histoire Naturelle (MNHN), Institut de Systématique, Évolution, Biodiversité (ISYEB), UMR 7205 CNRS-UPMC-EPHE, Sorbonne Universités, Paris, France; 2 Department of Biology and Agriculture, University of Central Missouri, Warrensburg, MI, USA

**Keywords:** Altitude, Biodiversity, Cicadomorpha, Fulgoromorpha, Elevational gradient

## Abstract

Malaise trap sampling of Hemiptera (Heteroptera; Auchenorrhyncha) was conducted at 500 m intervals along an elevational gradient from 200 m to 3,700 m on the east slope of Mount Wilhelm, Madang Province, Papua New Guinea. Hemiptera had a decrease in morphospecies richness and overall abundance with increasing elevation, however, the Heteroptera did not exhibit either pattern. A few species were relatively abundant at each elevation, whereas the majority of species were represented by ≤5 specimens. Morphospecies richness of Auchenorrhyncha, Cicadomorpha, Fulgoromorpha, Cicadellidae, Cixiidae, and Derbidae also decreased with increasing elevation but abundance decline was not significant due to the large number of specimens captured at 200 m relative to those captured at higher elevations. The percentage of Cicadomorpha specimens decreased with increasing elevation relative to that of the Fulgoromorpha which increased with increasing elevation. Environmental factors that may influence patterns of species richness along the elevational gradient are discussed.

## Introduction

The high organismic diversity of tropical rainforests has been the focus of numerous studies including those that document the diversity of selected taxa and others that seek to elucidate patterns. One type of pattern that emerges is the change in species richness and differences in the composition of insect communities with increasing elevation (Whittaker, 1952; Brühl, Mohamed & Linsenmair, 1999; Van Ingen, Campos & Andersen, 2008). Some studies found a decrease in species richness with increasing elevation (Hunter & Yonzon, 1992; Vázquez & Givnish, 1998) whereas some found the opposite—an increase in species richness with increasing elevation (Sanders, Moss & Wagner, 2003; Hodkinson, 2005). About half of the studies evaluated by Rahbek (2005) indicate that species richness increases, reaches a peak, then declines with increasing elevation, but this or other patterns can result from differences in spatial grain and sampling methodology (Janzen et al., 1976; Grytnes & Vetaas, 2002; Grytnes, Heegaard & Ihlen, 2006; Wiens et al., 2007; Guo et al., 2013). Suggested reasons for these patterns include: size of the habitat; isolation from similar communities; primary productivity as affected by temperature, length of the growing season and organism response to changing environmental conditions; and resource and habitat suitability (Hodkinson, 2005; McCain & Grytnes, 2010).

Papua New Guinea, which has the third largest expanse of tropical rainforest in the world (Brooks et al., 2006), provides opportunities to examine patterns of species richness and elevation in numerous taxa. Mount Wilhelm (4,509 m) has been the focus of studies of plant communities and elevational gradients (Brass, 1964; Hope, 1976; Munzinger et al., 2013), but few studies of the structure of phytophagous insect communities relative to their host plants have been conducted (Novotny et al., 2005; Dem, 2011).

Studies of taxa within the Hemiptera can provide useful insights about the ecological bases for distribution. Members of the Suborder Auchenorrhyncha are particularly suitable for study because, with the exception of some fungivores, almost all species are sap-feeders on xylem, phloem, or mesophyll as larvae and adults (Tonkyn & Whitcomb, 1987; Della Giustina, 1989; McKamey, 1999; Attié et al., 2008). Furthermore, they are basal heterotrophs which, relative to their host plant associations, have been treated as members of sap-feeding guilds (Denno, 1980), and many are monophagous or have a limited host plant range (Wilson et al., 1994). As well, measures of their species richness and diversity have been used as indicators of habitat quality —the “Auchenorrhyncha Quality Index” (Wallner, Molano-Flores & Dietrich, 2012; Spagnolo et al., 2014). However, relatively few studies have focused on the Auchenorrhyncha (McCoy, 1990; Novotný, 1992; Novotný, 1993; McKamey, 1999).

Studies of biodiversity and elevational gradients are “natural experiments” that can evaluate ecological theories on climate change as they are keys to understanding how changes in abiotic factors, especially temperature, can affect faunal and floral distribution (Guo et al., 2013; Sundqvist, Sanders & Wardle, 2013).

The focus of our study is to document the distribution of Auchenorrhyncha and Heteroptera along a rainforest elevational gradient, to determine the effect of elevation on species richness and abundance, and to discuss the factors affecting distribution.

## Materials and Methods

### Study area

**Figure 1:**
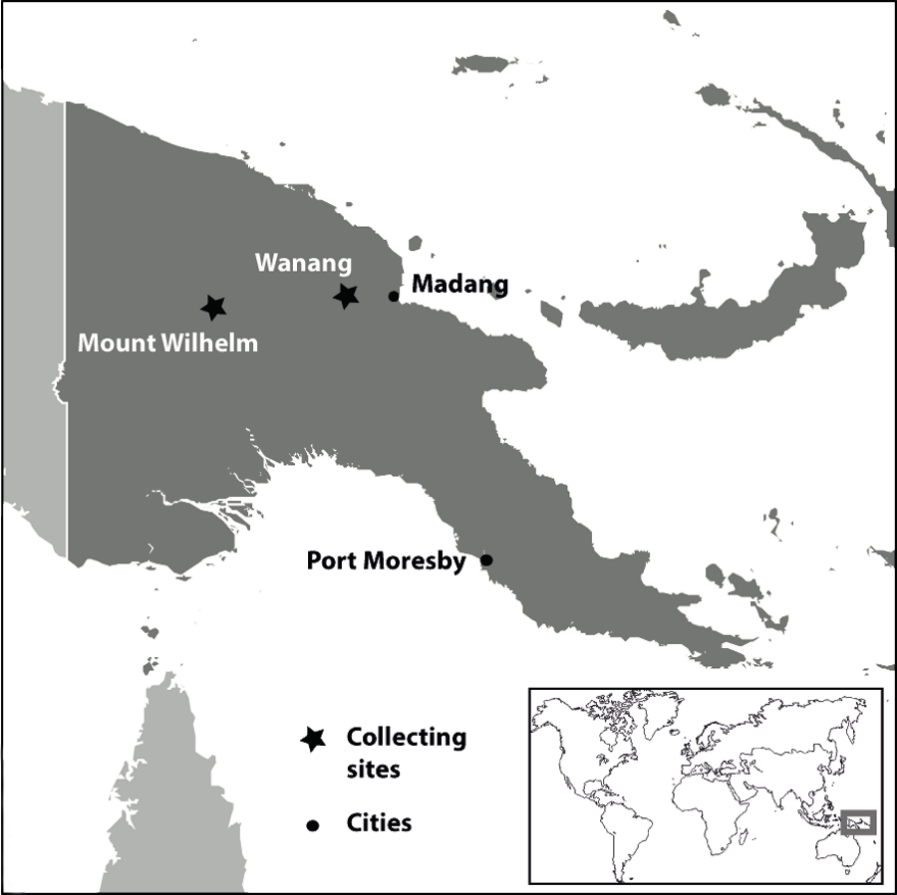
Location of collecting sites in Papua New Guinea.

The study was conducted along an elevational transect on the northeast aspect of Mount Wilhelm in Papua New-Guinea (Fig. 1). The transect followed the crests of the east slope of the mountain from 5°44′14.89″S, 145°19′56.13″E to 5°47′27.23″S, 145°3′29.58″E and began at 200 m elevation and extended to 3,700 m (Table 1), which represents the limit of the forest. The zonation of vegetation along the mountain slope (Hope, 1976) corresponds to changes in temperature and humidity. At elevations less than 1,000 m the tropical rainforest is dominated by Dipterocarpaceae, the average daily temperature fluctuates between 25 and 30 °C, and rainfall is greater than 4,000 mm/year. Between 1,000 m and 2,500 m, Lauraceae and Fagaceae are dominant and the average daily temperature ranges from 15 to 20 °C. From 2,500 m to 3,000 m, Podocarpaceae become increasingly abundant and the average daily temperature is ca. 12 °C. Above 3,000 m the sub-alpine vegetation is dominated by tree ferns, Cyatheaceae, the average daily temperature is ca. 8 °C, and rainfall is <3,400 mm/year (Table 2) (Hope, 1976; Munzinger et al., 2013; Duvot, 2013). Ninety-seven percent of the land in Papua new Guinea is owned by village communities. As such, they are important players in the preservation of the enormous biodiversity on their lands. Deforestation pressure is high but remote communities, height clans in total, from Wanang and Mt Wilhelm villages decided to be involved in the project and opted for conservation instead of logging. Thus, the collecting sites were chosen according to the possibilities to access them and their qualities. Even if the villagers have an certain impact on the primary forest, the selected sites were well preserved. The human pressure and damages on the primary vegetation were higher for the sites bellow 1,000 m.

**Table 1:** Location of the Malaise traps.

		Latitude	Longitude	Elevation
200 m	Plot A	5°44′23.63″S	145°19′47.07″E	293 m
	Plot B	5°44′27.71″S	145°19′45.79″E	333 m
	Plot C	5°44′41.24″S	5°44′41.24″S	375 m
	Plot D	5°44′14.89″S	145°19′56.13″E	214 m
700 m	Plot A	5°43′55.06″S	145°15′7.79″E	728 m
	Plot B	5°43′57.71″S	145°15′20.04″E	736 m
	Plot C	5°43′57.05″S	145°15′24.54″E	757 m
	Plot D	5°43′39.91″S	145°15′28.59″E	837 m
1,200 m	Plot A	5°43′15.15″S	145°16′10.07″E	1,188 m
	Plot B	5°43′15.68″S	145°16′13.09″E	1,201 m
	Plot C	5°43′15.24″S	145°16′17.28″E	1,223 m
	Plot D	5°43′16.93″S	145°16′13.10″E	1,199 m
1,700 m	Plot A	5°45′34.45″S	145°14′8.19″E	1872 m
	Plot B	5°45′35.68″S	145°14′5.02″E	1,874 m
	Plot C	5°45′39.30″S	145°13′24.72″E	1,885 m
	Plot D	5°45′11.56″S	145°14′13.32″E	1,614 m
2,200 m	Plot A	5°45′32.32″S	145°11′9.84″E	2073 m
	Plot B	5°45′36.64″S	145°11′10.53″E	2,070 m
	Plot C	5°45′39.70″S	145°11′9.72″E	2,066 m
	Plot D	5°45′26.25″S	145°11′0.29″E	2,134 m
2,700 m	Plot A	5°48′54.98″S	145°9′23.28″E	2,688 m
	Plot B	5°48′53.88″S	145°9′28.66″E	2,680 m
	Plot C	5°48′53.06″S	145°9′31.80″E	2,654 m
	Plot D	5°48′53.54″S	145°9′20.17″E	2,696 m
3,200 m	Plot A	5°48′24.11″S	145°4′22.52″E	3,180 m
	Plot B	5°48′26.71″S	145°4′25.08″E	3,076 m
	Plot C	5°48′25.00″S	145°4′19.70″E	3,182 m
	Plot D	5°48′4.65″S	145°4′8.61″E	3,361 m
3,700 m	Plot A	5°47′10.11″S	145°3′35.44″E	3,750 m
	Plot B	5°47′13.82″S	145°3′34.46″E	3,697 m
	Plot C	5°47′8.32″S	145°3′28.94″E	3,746 m
	Plot D	5°47′27.23″S	145°3′29.58″E	3,574 m

**Table 2:** Temperatures along the elevational gradient.

	<1,000 m	1,000–2,500 m	2,500–3,000 m	>3,000 m
Max. temperature (°C)	29.7	27.3	13.1	6.1
Min. temperature (°C)	24.8	15.3	9.7	10.4
Mean daily temperature (°C)	27.38	18.34	12.12	8.38

### Study material

We focused on collecting specimens of the hemipteran suborders Heteroptera and Auchenorrhyncha, although we also collected a few Sternorrhyncha (Table 3). The Auchenorrhyncha consists of the Fulgoromorpha (planthoppers) with 21 families, and the Cicadomorpha (leafhoppers, froghoppers, treehoppers, and cicadas) with 12 families (Cryan, 2005; Bourgoin, 2013; Soulier-Perkins, 2013), one of which, the Cicadellidae (leafhoppers), represented the majority of collected specimens.

**Table 3:** Species richness and abundance of Hemiptera collected along an elevational gradient on Mt. Wilhelm, Papua New Guinea.

	Number of	
Taxon	Morphospecies	Specimens
Heteroptera	46	217
Auchenorryncha		
Cicadomorpha		
Cicadellidae	303	2,544
Cicadellidae (larvae)	23	29
Cercopidae	11	19
Aphroporidae	1	1
Cicadidae	3	3
Membracidae	2	2
Fulgoromorpha		
Fulgoromoprha (larvae)	23	47
Cixiidae	53	179
Delphacidae	18	24
Derbidae	63	116
Achilidae	19	72
Meenoplidae	6	41
Dictyopharidae	2	2
Flatidae	5	6
Issidae	2	2
Fulgoridae	1	1
Ricaniidae	1	1
Sternorrhyncha		
Aleyrodoidea	3	3
Coccoidea	1	1
Psylloidea	8	8

### Sampling method

Sampling was conducted for 16 days, from 25 October to 10 November 2012 at eight sites placed every 500 m along the elevational transect on the east aspect of Mount Wilhelm. Four Malaise traps (Gibb & Oseto, 2006) were placed at random at each site; after placing the first Malaise trap the three others were set up every 100 m following the same contour line. After we observed numerous ants on the Malaise traps set up at 200 m, we established a ninth sampling site employing the same protocol at Wanang from 18 November to 4 December at 200 m in order to provide samples untouched by ants, if needed. The contents of each trap were collected each day by the parataxonomists from the Binatang research center and preserved in 90% ethyl alcohol and placed in a zip-lock bag. The material was sorted to family before being exported from Papua New Guinea under permit number 012297 granted by the Department of Environment and Conservation of Papua New Guinea.

All specimens were examined at the Muséum National d’Histoire Naturelle (Paris, France) using a Leica MZ16 stereo microscope and identified to morphospecies which is a useful means of identifying large numbers of specimens for ecological studies (Oliver & Beattie, 1993; New, 1998). Photographs using a Canon EOS 50D of representatives of each morphospecies were taken in order to facilitate identification. Recognition of morphospecies was based on morphological characters of the head, thorax, abdomen and legs. Each morphospecies is vouchered accordingly to the elevation, the trap and the day it was collected (see Supplemental Information).

### Data analysis

The relationship between elevation and morphospecies richness and abundance was examined using Pearson product moment correlations (Roscoe, 1975) and the Shannon–Weiner Diversity Index (Krebs, 1989). A factorial correspondence analysis (FCA) was also used (ade4 package, Dray & Dufour, 2007) in R, version 3.0.2 (R Development Core Team, 2013) to study the arrangement of morphospecies along the elevational gradient (Benzécri, 1964). For this multivariate analysis, each morphospecies was coded 1 if present and 0 if not; each line represented a trap and each column a morphospecies.

## Results

In total, 4,205 specimens were sorted and 713 morphospecies identified; 3,318 specimens representing 596 morphospecies were from the collecting stations on Mount Wilhelm, the remainder were collected in Wanang and used as reference material for our study (Table 3; Supplemental Information).

### Morphospecies distribution

A succession of morphospecies was observed along the elevational gradient. Each morphospecies was rarely collected at more than one elevation (Table 4); one cicadellid species was collected from 200 to 2,200 m but in diminishing numbers. From 6% to 19% of the morphospecies consisted of more than five specimens at any given elevation whereas 81% to 94% of morphospecies were represented by five or fewer specimens (Table 5).

**Table 4:** Number of identical morphospecies collected at each elevation (m).

	Elevation (m)							
	200	700	1,200	1,700	2,200	2,700	3,200	3,700
200	–	22	5	2	1	0	0	0
700	22	–	9	3	0	0	0	0
1,200	5	9	–	4	0	1	0	0
1,700	2	3	4	–	6	2	2	0
2,200	1	0	0	6	–	4	2	0
2,700	0	0	0	2	4	–	8	0
3,200	0	0	0	2	2	8	–	5
3,700	0	0	0	0	0	0	5	–

**Table 5:** Relative morphospecies richness (%) at each elevation (m).

	Elevation (m)							
	200	700	1,200	1,700	2,200	2,700	3,200	3,700
% >5	14	8	6	11	7	8	8	19
% ≤5	86	92	94	89	93	93	92	81
*N*	163	133	69	72	81	40	52	21

**Figure 2:**
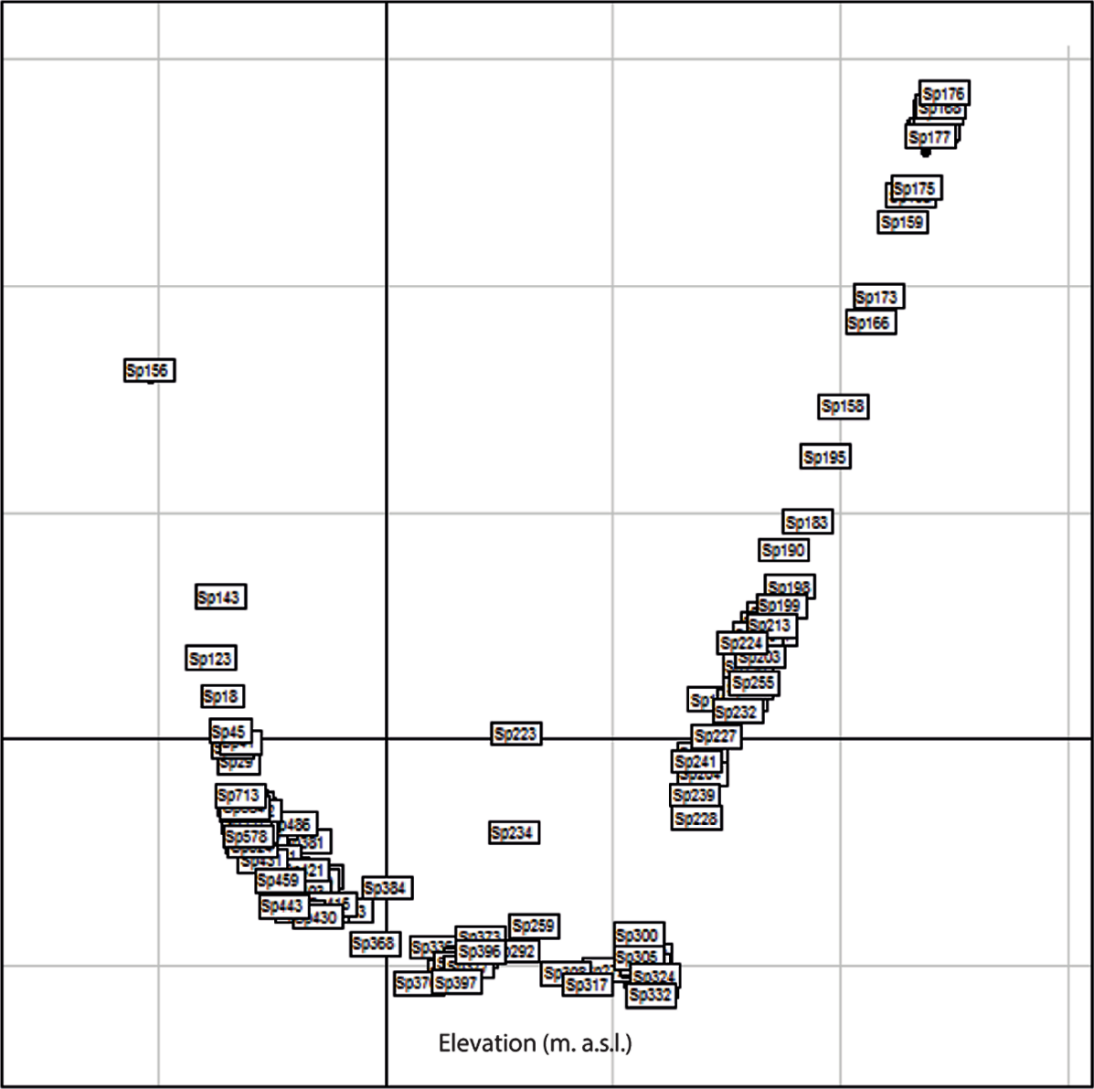
Factorial Correspondence Analysis (FCA) of the arrangement of morphospecies (*x*-axis) along the elevational gradient. Each block corresponds to a morphospecies.

**Figure 3:**
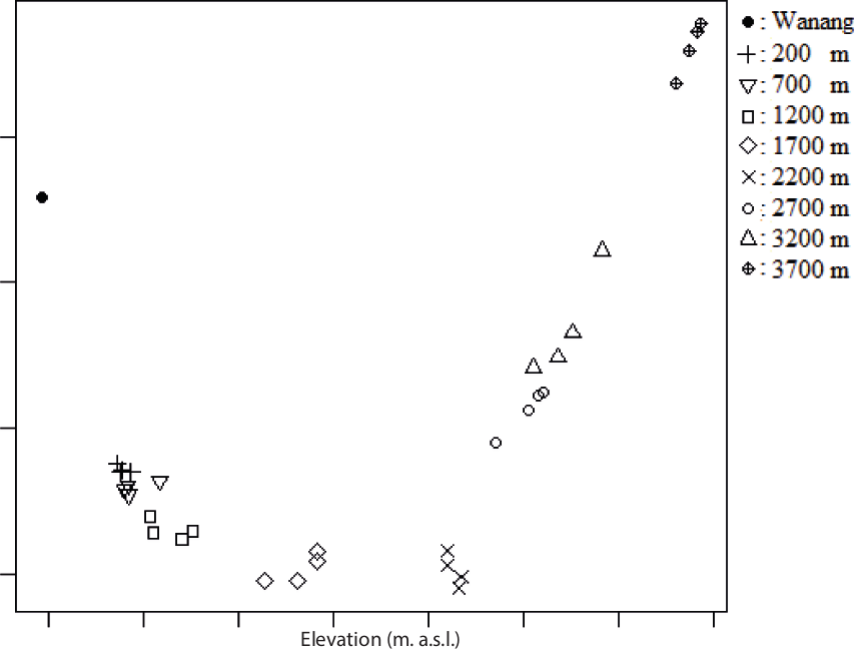
Factorial Correspondence Analysis (FCA) of the arrangement of collecting traps (*x*-axis) along the elevational gradient. Groupings indicate that traps at the same elevation had shared morphospecies.

Analysis via Factorial Components Analysis suggested that there was a succession of morphospecies along the elevational gradient and that there were few species that occurred at more than one elevation (Table 4; Figs. 2 and 3).

### Morphospecies richness

**Figure 4:**
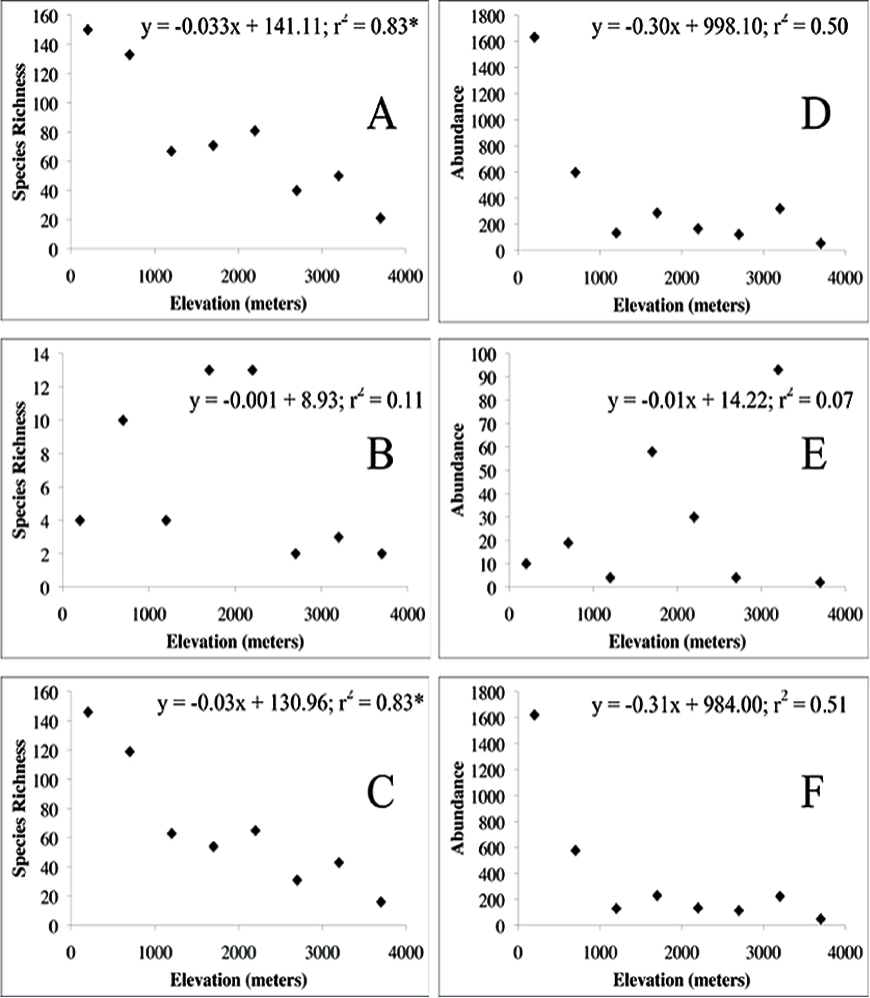
Elevation, species richness, and abundance. (A–C) Elevation and species richness. (D–F) Elevation and abundance. (A, D) Hemiptera. (B, E) Heteroptera. (C, F) Auchenorrhyncha. ^∗^*p* < 0.05.

Overall species richness declined with increasing elevation (Fig. 4A; Table 6). Examining the relationship between elevation and each taxon revealed that there was no relationship between elevation and Heteroptera species richness (Fig. 4B). A pattern of decreasing richness with increasing elevation was found for the Auchenorrhyncha (Fig. 4C) as well as for the Cicadomorpha, Fulgoromorpha, Cixiidae, and Derbidae (Table 6).

**Table 6:** Relationship among morphospecies richness and abundance and elevation (m).

Taxon	Regression^a^	*r* ^2^	Significance
**Secies richness**			
Hemiptera	*y* = − 0.033*x* + 141.11	0.83	^*^
Heteroptera	*y* = − 0.001 + 8.93	0.11	–
Auchenorrhyncha	*y* = − 0.03*x* + 130.96	0.83	^*^
Cicadomorpha	*y* = − 0.02*x* + 86.14	0.72	^*^
Fulgoromorpha	*y* = − 0.01*x* + 45.75	0.92	^*^
Cicadellidae	*y* = − 0.022*x* + 83.66	0.72	^*^
Cixiidae	*y* = − 0.003*x* + 12.98	0.71	^*^
Derbidae	*y* = − 0.004*x* + 17.14	0.79	^*^
**Abundance**			
Hemiptera	*y* = − 0.30*x* + 998.10	0.50	–
Heteroptera	*y* = − 0.01*x* + 14.22	0.07	–
Auchenorrhyncha	*y* = − 0.31*x* + 984.00	0.51	–
Cicadomorpha	*y* = − 0.271*x* + 850.63	0.52	–
Fulgoromorpha	*y* = − 0.0371*x* + 133.37	0.43	–
Cicadellidae	*y* = − 0.27*x* + 846.45	0.52	–
Cixiidae	*y* = − 0.012*x* + 46.46	0.28	–
Derbidae	*y* = − 0.005*x* + 24.71	0.34	–

**Notes.**

a Pearson Product Moment Correlation.

* *p* < 0.05.

### Morphospecies abundance

The number of specimens captured by the traps appeared to decrease with increasing elevation; however, the correlation was not significant (Fig. 4D; Table 6). As with species richness, no relationship was found between elevation and the abundance of Heteroptera (Fig. 4E). Similarly, species abundance appeared to decrease with increasing elevation for the Auchenorrhyncha (Fig. 4F), the Cicadomorpha, Fulgoromorpha, Cicadellidae, Cixiidae, and Derbidae (Table 6); however, the correlations were not significant. The large number of specimens captured at 200 m relative to those captured at higher elevations resulted in correlations that were not significant.

Shannon–Weiner Diversity Indices. The highest diversity indices were at the two lowest elevations, which corresponds to the patterns of morphospecies richness and abundance (Figs. 4A and 4D; Table 7). Between 1,200 m and 3,700 m the diversity indices increased then declined.

**Table 7:** Shanon–Weiner diversity indices and elevation (m).

Elevation	Shannon Wiener index
200	2.558
700	1.529
1,200	0.519
1,700	1.252
2,200	1.293
2,700	1.092
3,200	1.161
3,700	0.922

### Cicadomorpha and Fulgoromorpha species richness

**Figure 5:**
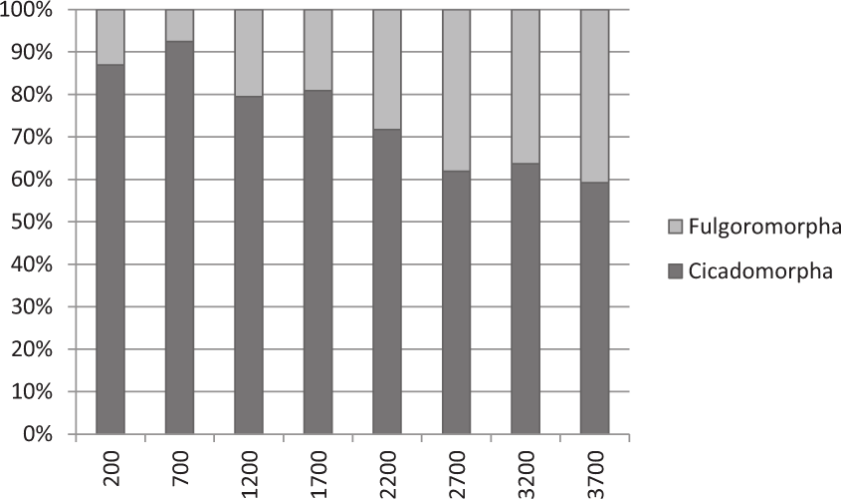
Relative percentages of the number of Cicadomorpha and Fulgoromorpha at each elevation (m). (One morphospecies of cicadellid was removed from analysis).

As indicated above, the Auchenorrhyncha species richness decreased with increasing elevation which led us to further examine species richness patterns in the Cicadomorpha and Fulgoromorpha. Comparison of the proportions of Cicadomorpha relative to Fulgoromorpha suggested that the proportion of Fulgoromorpha increased with increasing elevation. The number of cicadomorph specimens collected at 3,200 m appears to refute this suggestion; however, one cicadellid morphospecies represented 73.5% of all Cicadomorpha collected at this elevation. After removing this morphospecies from the analyses, we found that the Fulgoromorpha represented an increasing proportion of Auchenorrhyncha from ca. 10% at 200 m to ca. 40% at 2,700 m (Fig. 5).

## Discussion

### Morphospecies distribution, richness, and abundance

Factorial Components Analysis indicated that there was a sequence of morphospecies corresponding to the elevational gradient of Mount Wilhelm. There was a negative correlation of species richness and elevation for the Hemiptera. There was no relationship between species richness and elevation for the Heteroptera, which may be because of weak association with plant taxa as some were polyphagous and others predaceous (Schuh & Slater, 1995). The Auchenorrhyncha, Cicadomorpha, Fulgoromorpha, Cicadellidae, Cixiidae, and Derbidae all had the highest species richness at the lowest elevation and richness generally decreased with increasing elevation. As noted above, Auchenorrhyncha species are phytophagous (Mitter, Farrell & Wiegmann, 1988), Cicadomorpha are phloem, xylem, or mesophyll feeders, Fulgoromorpha feed on phloem or fungi (Tonkyn & Whitcomb, 1987; Wilson et al., 1994) and numerous species of these taxa are mono- or oligophagous (Wilson et al., 1994; Attié et al., 2008). The greatest plant diversity occurred at the lowest elevations with three other plant communities occurring at ca. 1,000, 2,500, and 3,000 m in elevation, respectively (Hope, 1976; Hodkinson, 2005). Differences in plant diversity and communities are likely factors that explain, in part, the observed elevation gradient in species richness.

Abiotic factors that may explain the distribution of the hemipteran taxa include the climatic changes that occur with increasing elevation. Temperature and rainfall decrease significantly from 25–30 °C and ca. 4,000 mm/year at lower elevations to <8 °C and ca. 3,400 mm/year at the highest elevation. These factors directly affect insect development and survival and correspond to the zonation of the vegetation which indirectly affects the distribution of hemipterans (McCain & Grytnes, 2010; Régnière et al., 2012; Savopoulou-Soultani et al., 2012).

Species richness decreased with increasing elevation which is similar to patterns observed in several studies (Hunter & Yonzon, 1992; Vázquez & Givnish, 1998). The abiotic factors cited above can explain, in part, the decrease in species richness with increasing elevation that we observed. Although species richness generally decreased with elevation, there were slight increases in richness at 2,200 m and 3,200 m (Figs. 4A and 4C). An increase in hemipteran species richness at these two elevations was also observed by Dem (2011). This hump-shaped pattern was also inferred from the Shannon–Weiner Diversity Indices between 1,200 and 3,700 m (Table 7) and has been found in more detailed studies of other insect taxa (McCoy, 1990; Brehm, Colwell & Kluge, 2007). Slightly higher species richness at these elevations could correspond to regions where plant communities from lower and higher elevations intergrade, or it could be a response to the distribution of insectivores (Sam et al., 2014).

At every taxonomic level evaluated, there was no correlation between abundance and elevation (Figs. 4D and 4F) with very large numbers of specimens captured at the lowest elevations and substantially fewer at higher elevations (except for the Heteroptera). For the Auchenorrhyncha species, abundance increased at 2,200 m and 3,200 m (Fig. 4F), which could correspond to regions where plant communities from lower and higher elevations intergrade.

### Species richness and elevation and sampling methodology

Malaise trap sampling is a very effective means of sampling a portion of an insect community, but as with any single collecting technique, it cannot provide a complete survey of the insect fauna (Leather & Watt, 2005; Ozanne, 2005). Apterous and brachypterous insects, those that do not leave their host plants, and those that live in the forest canopy are less likely to be captured in the traps. Placement of traps in areas where vegetation is too dense or too sparse will affect capture rate (Ozanne, 2005). This was addressed in our study by random placement of the four traps at each site with the expectation that traps placed in areas with an adequate representation of the hemipteran fauna will compensate for those placed in less suitable areas (Smith, 2013).

Papua New Guinea has a tropical climate with alternating wet and dry seasons. Accurate sampling of hemipterans is a function of the linkage of life cycles to this seasonality. We collected for a short period of time; however, it was done during the optimum collecting period for planthoppers and leafhoppers (Novotný & Basset, 1998).

### Cicadomorpha and Fulgoromorpha species richness and abundance

The Cicadomorpha and Fulgoromorpha were the dominant taxa in terms of species richness and abundance (Table 3). The relative percentages of the numbers of Cicadomorpha and Fulgoromorpha collected at each elevation (Fig. 5) indicated that the proportion of Cicadomorpha decreased with increasing elevation whereas the Fulgoromorpha increased. However in order to show this general pattern, one morphospecies of cicadellid was removed from the analysis. Its outbreaks in the Malaise traps tended to mask the inversely proportional tendencies observed between Cicadomorpha and Fulgoromorpha along the altitudinal gradient.

In the Cicadomorpha, the Cicadellidae consisted of 80% of all Hemiptera collected. In addition, only few Cercopidae, one Aphrophoridae, two Membracidae, and three Cicadidae were collected.

In the Fulgoromorpha, the families with the highest species richness and abundance were the Achilidae, Cixiidae, and Derbidae. The remaining six families included substantially fewer morphospecies and individuals (Table 3). The Achilidae have been associated with species in 27 families of plants, the Cixiidae with species in 88 families, and the Derbidae with species in 28 families (Wilson et al., 1994; Bourgoin, 2013). Three of the plant families associated with Cixiidae and Derbidae, Cyatheaceae, Podocarpaceae and Fagacae, are major components of the three highest plant communities along our elevational transect.

Also, these three planthopper families, which represented 79% of morphospecies (*N* = 170) and 90% (*N* = 404) of individual planthoppers, have larvae that feed underground on plant roots (Cixidae) or, it is presumed, fungal hyphae (Achilidae, Derbidae) (Wilson et al., 1994).

Our inventory of Hemiptera along an elevational gradient on Mt. Wilhelm resulted in finding no pattern of morphospecies distribution and abundance among Heteroptera but declines in morphospecies richness with increasing elevation in the Auchenorrhyncha and its subgroups. The decreasing proportion of Cicadomorpha morphospecies relative to Fulgoromorpha with increasing elevation may be due to differences in host plant communities or larval habitats and therefore warrants further study.

## Supplemental Information

10.7717/peerj.978/supp-1 Supplemental Information 1 Morphospecies inventory for each station and each day

## Additional Information and Declarations

### Field Study Permissions

The following information was supplied relating to field study approvals (i.e., approving body and any reference numbers): Department of Environment and Conservation of Papua New Guinea Permit no 012297.
